# A lightweight dual branch masking network for environmental sound classification

**DOI:** 10.1038/s41598-025-33636-w

**Published:** 2025-12-31

**Authors:** Guorong Chen, Bao Zhang, Zhikang Ding, Ke Xiao, Pengyu Guan, Xianghan Xiao, Xiaoqiang Wang, Haixin Yi, Hong Hu, Weijie Zhang

**Affiliations:** 1 School of Computer Science and Engineering, Chongqing University of Science and Technology, Chongqing, 401331 China; 2 Chongqing Institute of Intelligent Mathematics and Autonomous Intelligence, Shapingba District, 401127 Chongqing, China; 3 PetroChina Sichuan Luzhou Shale Gas Exploration and Development Co., Ltd, Neijiang, 641000 China

**Keywords:** Engineering, Mathematics and computing

## Abstract

Environmental sound classification (ESC) is crucial for applications such as intelligent surveillance, urban acoustic monitoring, and human-computer interaction. Although deep neural networks (DNNs) have significantly improved ESC performance, these methods often rely on large models and extensive pretraining, making them difficult to deploy in resource-constrained environments. Some existing lightweight models, while having fewer parameters, still suffer from limited representational capacity, leading to suboptimal generalization, especially in low-data scenarios. To address these challenges, we propose SpectroMaskNet, a compact dual-branch architecture. This design integrates global-local attention mechanisms with block-masked spectrogram augmentation, allowing the model to capture both long-term temporal dependencies and fine-grained spectral features. This enhances robustness and generalization, particularly in data-scarce situations. Experimental results on four benchmark datasets–ESC-10, ESC-50, UrbanSound8K, and SpeechCommandV2–demonstrate that SpectroMaskNet achieves accuracies of 97.50%, 95.50%, 96.32%, and 96.52%, respectively, outperforming existing lightweight baselines without requiring large-scale pretraining. Furthermore, the model maintains low computational complexity, making it well-suited for real-world ESC applications that demand efficiency and scalability.

## Introduction

Environmental sound classification (ESC) has attracted increasing attention in recent years due to its wide range of applications in intelligent surveillance, urban acoustic monitoring, wildlife conservation, and human–computer interaction. Unlike speech or music classification, ESC involves a wide variety of sound events that often overlap, making accurate recognition more challenging. In recent years, advances in deep neural networks such as convolutional neural networks (CNNs), attention mechanisms, and Transformer-based architectures have significantly improved ESC performance on multiple benchmark datasets.

Nevertheless, many high-performing ESC systems rely on large-scale architectures with tens of millions of parameters and require pretraining on massive audio corpora followed by fine-tuning on the target dataset, such as Efficient Audio Transformer (EAT)^1^ ( 86M parameters), Audio Spectrogram Transformer (AST)^2^ ( 88M parameters), PaSST^3^ ( 85M parameters), and various audio classification models adapted from the Vision Transformer (ViT)^4^ architecture (typically 80M-100M parameters). While these methods can achieve state-of-the-art accuracy, they are computationally expensive and unsuitable for deployment in resource-constrained environments such as embedded devices or edge computing platforms. In addition, lightweight models with fewer than 5 million parameters still suffer from limited representational capacity and are prone to overfitting when trained directly on small-scale datasets, leading to suboptimal performance.

To address these issues, we propose **SpectroMaskNet**, a lightweight method for environmental sound classification. First, we introduce a block-masked spectrogram augmentation strategy that randomly masks local regions in the time–frequency representation to simulate scenarios of partial information loss, thereby enhancing robustness and improving generalization in low-data regimes. Second, we design a compact dual-branch architecture that integrates global–local attention mechanisms, with one branch capturing long-term temporal dependencies and the other focusing on fine-grained spectral features, enabling complementary feature extraction and boosting overall classification accuracy. Extensive experiments on four widely used benchmark datasets–ESC-10^5^, ESC-50^5^, UrbanSound8K^6^, and SpeechCommandV2^7^–show that SpectroMaskNet outperforms existing lightweight baselines without requiring large-scale pretraining. These results demonstrate the method’s strong potential for efficient and practical environmental sound classification tasks.

## Related work

### Deep learning in ESC

Early research on environmental sound classification (ESC) primarily relied on handcrafted features and conventional classifiers. Cowling and Sitte^8^ systematically evaluated various time–frequency statistical features, such as Mel-frequency cepstral coefficients (MFCCs^9^), zero-crossing rate, and spectral centroid, in combination with classifiers like kNN^10^, SVM^11^, and decision trees^12^. Their results demonstrated that these handcrafted features exhibited reasonable discriminative ability under low-noise conditions; however, performance degraded considerably in the presence of strong background noise or overlapping sources, revealing limited generalization capacity. Building upon this, Chu et al.^13^ proposed an ESC framework integrating short-time Fourier transform (STFT)^14^ features with Gaussian mixture models (GMMs)^15^, which improved accuracy through time–frequency modeling. Nonetheless, these approaches heavily depended on handcrafted feature engineering and lacked the ability to model non-stationary and long-term temporal dependencies.

The emergence of deep learning marked a paradigm shift in ESC research. Piczak^16^ was the first to apply 2D convolutional neural networks (CNNs) to ESC by treating log-Mel spectrograms as images, achieving substantial improvements on ESC-10 and ESC-50 datasets (about 80% and 64%, respectively). However, due to the shallow network structure, its feature extraction capability remained limited and sensitive to noise and domain variations. To address these issues, Salamon et al.^17^ proposed deep convolutional neural networks (DCNNs) with data augmentation techniques such as time-shifting, noise injection, and dynamic range compression, boosting UrbanSound8K accuracy to 79% while effectively reducing overfitting. Despite these gains, DCNNs still involved large parameter counts and high computational costs, making them impractical for low-resource applications.

To enhance temporal modeling, Çakir et al.^18^ introduced convolutional recurrent neural networks (CRNNs), which combined the local pattern extraction capability of CNNs with the long-term dependency modeling of RNNs, and incorporated a weighted pooling mechanism to improve multi-label ESC performance. Although these models achieved leading results in DCASE challenges, their success on small-scale datasets still relied heavily on data augmentation and transfer learning.

Furthermore, Hershey et al.^19^ utilized Google’s large-scale AudioSet to pretrain CNN-based models and subsequently fine-tuned them on ESC tasks, achieving substantial performance improvements across multiple datasets. This work established the dominant paradigm of “large-scale pretraining followed by fine-tuning”. However, such methods required tens of millions of parameters and considerable computational resources, imposing high deployment costs and limiting reproducibility without access to large-scale pretraining data.

### Attention mechanisms in sound classification

In recent years, attention mechanisms have been widely applied in various fields, including image restoration and sound classification. By capturing key information across different time and frequency scales, attention mechanisms can effectively enhance model performance by focusing on important parts of the data and improving the understanding of global context.

For example, the Encoder-Free Multiaxis Physics-Aware Fusion Network (EMPF-Net)^20^ for remote sensing image dehazing employs a multi-axis attention mechanism to integrate features without using a traditional encoder-decoder architecture. This approach provides insights for the application of attention mechanisms in sound classification, particularly when fine-grained feature extraction is required from noisy or incomplete audio signals. Another relevant work, All-in-One Weather-Degraded Image Restoration via Adaptive Degradation-Aware Self-Prompting Model (ADSM)^21^, combines self-prompting mechanisms with diffusion models to restore images with various weather degradations. This method demonstrates how adaptive mechanisms can handle different types of degradation, offering valuable references for addressing environmental noise and complex backgrounds in sound classification.

Additionally, the Dynamic Feature Attention Network (DFA-Net)^22^, which is designed for remote sensing image dehazing, introduces dynamic attention mechanisms for cross-layer interaction, effectively capturing multi-scale information in images. A similar mechanism could be applied to sound classification, enhancing the modeling of multi-scale audio features, especially when handling complex audio signals, allowing for better integration of features from different layers.

These studies show that attention mechanisms not only help models focus on local features but also enhance the understanding of global context. In sound classification tasks, they are crucial for extracting time-frequency information and feature fusion. In this work, we draw upon these attention mechanisms and combine global and local information to improve accuracy and robustness in sound classification.

### Transformer-based models

Transformer-based architectures have made significant progress in environmental sound classification (ESC) in recent years. Gong et al.^2^ proposed the Audio Spectrogram Transformer (AST), the first work to directly adapt the Vision Transformer (ViT) architecture from the image domain to audio. AST treats log-Mel spectrograms as 2D images by dividing them into fixed-size patches and feeds the resulting patch sequence into a standard Transformer encoder, eliminating the need for convolutional layers. AST achieved state-of-the-art results on both ESC-50 and AudioSet datasets, demonstrating the Transformer’s strong ability to model long-range dependencies and global context. Additionally, AST leverages large-scale pretraining on AudioSet followed by fine-tuning on ESC datasets, which significantly improves generalization performance. However, the model contains over 85 million parameters, resulting in high computational cost and posing major challenges for deployment on edge devices or resource-constrained platforms.

To further improve both efficiency, Chen et al. proposed the Efficient Audio Transformer (EAT)^1^, which incorporates several key enhancements to the AST framework. First, EAT introduces a more effective spectrogram masking strategy, selectively masking non-contiguous time steps and frequency bands to improve robustness and training efficiency. Second, it adopts a teacher–student training paradigm, where knowledge from a larger model is distilled into a smaller student network, enhancing generalization under limited resources. Experimental results show that EAT achieves 96.0% accuracy on the ESC-50 dataset, outperforming conventional CNNs and Transformer models without masking strategies. Nevertheless, EAT still contains around 80 million parameters and remains heavily dependent on large-scale pretraining using datasets like AudioSet, which limits its applicability in real-time or low-resource deployment scenarios.

### Limitations of lightweight models

To mitigate the high computational cost of large Transformer-based architectures in environmental sound classification (ESC), various lightweight convolutional neural network (CNN) models have been proposed to balance efficiency and performance. For example, Mohaimenuzzaman et al. proposed ACDNet^23^, a resource-efficient model designed for extremely constrained devices, which achieves 87.1% accuracy on the ESC-50 dataset with only  1 million parameters and can be compressed further with minimal performance loss. Guzhov et al. introduced ESResNet^24^, a ResNet-based architecture adapted from the visual domain, and achieved 91.5% accuracy on ESC-50, demonstrating that visual-domain backbones can be effectively transferred to audio tasks. While these models exhibit strong parameter efficiency, their performance still lags behind that of large pretrained Transformer-based models.

To further improve the performance of lightweight models, Kong et al. proposed SSAST^25^, a model based on self-supervised pretraining with architectural simplifications. It contains approximately 6 million parameters and achieves 88.8% accuracy on ESC-50. However, SSAST heavily relies on pretraining strategies, and its performance drops significantly without access to large-scale audio corpora.

Overall, the underperformance of lightweight models on small-scale datasets is primarily due to their limited representational capacity. CNNs often focus on local time–frequency patterns and struggle to capture long-term dependencies or global contextual features. Under low-data regimes, these models are also prone to overfitting, especially in complex acoustic environments, leading to poor generalization. Furthermore, to reduce parameter counts, many lightweight models compromise on network depth or channel width, which restricts their feature modeling ability. Meanwhile, real-world deployment scenarios (e.g., edge devices, embedded systems, real-time acoustic sensors) demand models with low latency, low power consumption, and high portability–requirements that traditional large-scale models often fail to meet.

## Methods

### Overview of network architecture

Although deep neural networks have greatly advanced the field of environmental sound classification (ESC), we observed that conventional architectures are highly prone to overfitting when trained on small-scale datasets, while large models with tens of millions of parameters impose substantial computational costs, making them impractical for deployment on resource-constrained devices. To address this dual challenge, we adopted ResNet-SE as the baseline model and introduced a SpecAugment-based data augmentation strategy, which includes adding Gaussian noise to audio signals as well as applying time and frequency masking operations. These augmentation techniques effectively alleviated overfitting; however, experimental results on the ESC-50 dataset showed that the classification accuracy on both the training and validation sets decreased by approximately 2.5% compared with the baseline, indicating that traditional data augmentation methods, while providing regularization benefits, may simultaneously weaken feature integrity.

**Fig. 1:**
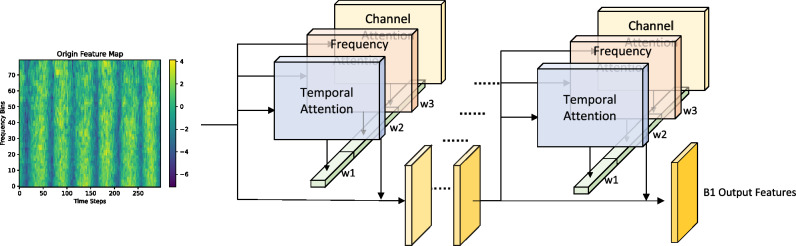
The figure illustrates the stacking architecture of multiple GlobalAttention modules within the global branch. Each module consists of three synergistic sub-branches–channel attention, temporal attention, and frequency attention–that collaboratively model feature interactions across multiple dimensions. The output of each upper module serves as the input to the next, enabling a progressive transition from low-level local representations to high-level global semantic features. The example shows two consecutive GlobalAttention blocks, with the final output denoted as B1_Output_Features.

**Fig. 2:**
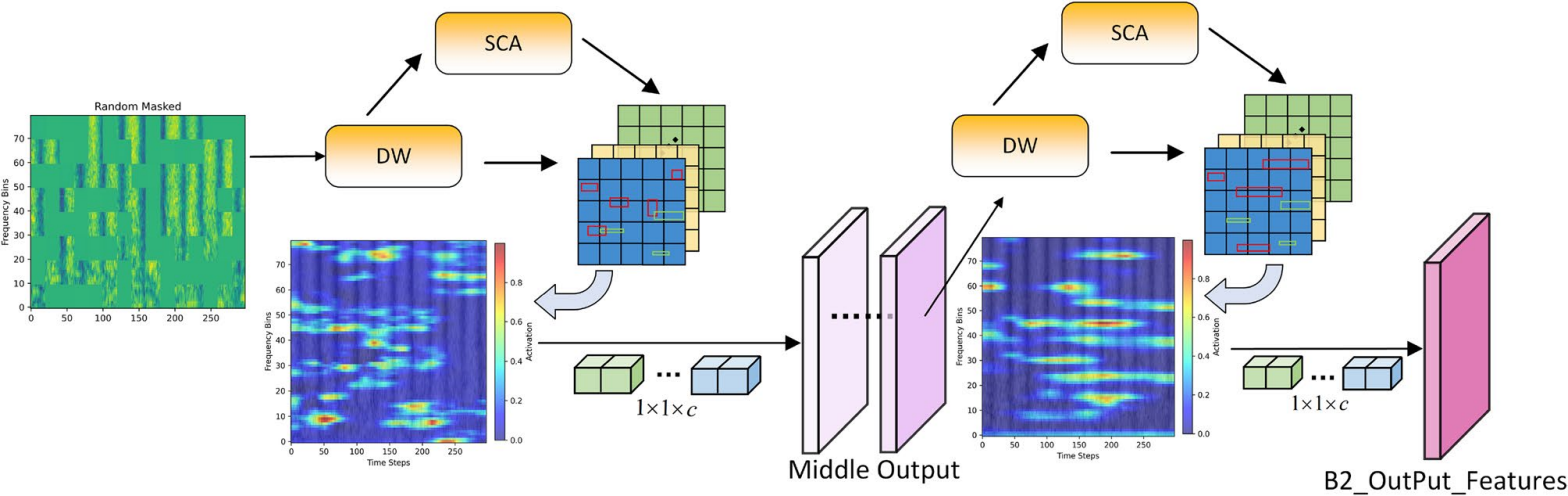
The figure illustrates the overall structure of the LocalAttention module. The input spectrogram first undergoes random block masking to enhance robustness.

To improve model performance while maintaining strong regularization capability, we propose a lightweight asymmetric dual-branch architecture, termed SpectroMaskNet, which integrates a learnable cross-attention masking mechanism within a global–local feature learning framework. The model consists of two complementary branches that process different forms of input. One branch (as illustrated in Fig. 1) receives the complete FBank feature map and is responsible for capturing holistic global time–frequency patterns. The other branch (also shown in Fig. 2) takes as input a masked version of the FBank feature map, where the masking ratio and block size are no longer predefined constants but are instead formulated as learnable parameters. These parameters are initialized randomly and optimized jointly with the network through backpropagation, enabling the model to dynamically adjust the masking strength and masked region size during training. This adaptive masking mechanism allows the local branch to continuously refine its focus on informative spectral regions, thereby enhancing robustness under partial or degraded information conditions.

The key innovation of SpectroMaskNet lies in its cross-attention allocation strategy. Unlike conventional dual-branch models that employ identical attention mechanisms in both paths, SpectroMaskNet assigns distinct attentional roles to each branch: the branch processing the complete spectrum utilizes a global attention mechanism to model contextual dependencies, whereas the branch processing the masked spectrum adopts a local attention mechanism to extract fine-grained spectral features. The outputs of the two branches are fused through a learnable weighting parameter $\lambda$, enabling the network to dynamically balance the importance of global and local feature representations during training.

#### Core parameters of each stage

The dual branches (global/local) of SpectroMaskNet both adopt a 4-stage stacked structure, with a unified layer configuration of [2, 3, 3, 2] (representing the number of attention modules contained in each stage) and corresponding convolutional channel dimensions of [16, 32, 48, 64]. The specific implementation details are as follows:

1. **Stage 1**: Consists of 2 attention modules (GlobalAttention for the global branch / LocalAttention for the local branch). The input channel is converted from 1 to 16, with no downsampling (stride=1). The convolutional kernel size is $3 \times 3$ (local branch) / $7 \times 7$ (global branch), and padding=1 is used to maintain consistent feature map size.
2. **Stages 2–3**: Each stage contains 3 attention modules. The channel dimensions are transformed from 16$\rightarrow$32 (Stage 2) and 32$\rightarrow$48 (Stage 3), respectively. Downsampling is implemented via convolution with stride=2, which halves the feature map size.
3. **Stage 4**: Comprises 2 attention modules, with the channel dimension converted from 48 to 64 and no downsampling. The output feature map is compressed into a 64-dimensional vector through Global Average Pooling (GAP).

#### Internal details of modules

- The GlobalAttention module in the global branch includes three parallel attention mechanisms (channel, temporal, and frequency attention). Dimension reduction (reduction=8) and restoration are achieved via $1 \times 1$ convolution, and the attention weights are fused through element-wise multiplication.
- The LocalAttention module in the local branch adopts $7 \times 7$ Depthwise Separable Convolution (DSC) to expand the receptive field, combined with Simplified Channel Attention (SCA), Local Feature Enhancement (LFE), and a gating mechanism. All modules are embedded with residual connections and GroupNorm normalization.

#### Feature fusion strategy

The feature fusion of the dual branches adopts a learnable weighted strategy, with the formula expressed as:

1 $$\begin{aligned} F = \lambda \cdot F_G + (1 - \lambda ) \cdot F_L \end{aligned}$$

where $F_G$ denotes the output feature of the global branch, $F_L$ represents the output feature of the local branch, and $\lambda$ is a learnable weight parameter. The specific implementation details are as follows:The initial value of $\lambda$ is set to 0.5. During training, $\lambda$ is jointly updated with other model parameters via the Adam optimizer, using the same learning rate as the main model ($1 \times 10^{-4}$). To avoid weight bias toward a single branch, the value range of $\lambda$ is constrained by L2 regularization. In actual training, $\lambda$ converges to $0.52 \pm 0.03$.

### Global branch design

To achieve comprehensive global modeling of audio features, the proposed **GlobalAttention module** introduces a multi-dimensional synergistic attention mechanism that simultaneously models feature interactions along the channel, temporal, and spectral dimensions, thereby enhancing the integrity and discriminative power of the learned representation. The module consists of three parallel attention branches that generate the channel attention map $A_c$, temporal attention map $A_t$, and frequency attention map $A_f$, respectively. In the channel branch, the input feature $X \in \mathbb {R}^{C\times T\times F}$ is first processed by global average pooling (GAP) to compress the temporal and frequency dimensions, yielding a compact channel descriptor:

2 $$\begin{aligned} z_c = \frac{1}{T \times F} \sum _{t=1}^{T} \sum _{f=1}^{F} X_{c,t,f}. \end{aligned}$$

Then, two consecutive $1\times 1$ convolutional layers with reduction and expansion operations are employed to compute channel importance weights:

3 $$\begin{aligned} A_c = \sigma \big ( W_2 \, \delta (W_1 z_c) \big ), \end{aligned}$$

where $W_1$ and $W_2$ represent the dimensionality reduction and expansion transformations, respectively, $\delta (\cdot )$ denotes the ReLU activation, and $\sigma (\cdot )$ denotes the Sigmoid function. This structure effectively balances expressive power and parameter efficiency, playing a key role in the lightweight design of the model. The temporal and frequency attention branches adopt a symmetric design: adaptive pooling is performed along the frequency and temporal dimensions, respectively, to preserve complete temporal and spectral structures. One-dimensional convolutions are then applied to capture dynamic temporal variations and discriminative spectral-band features, producing $A_t$ and $A_f$. Finally, the three attention maps are fused via element-wise multiplication to obtain the globally weighted feature representation:

4 $$\begin{aligned} X' = X \odot (A_c \otimes A_t \otimes A_f), \end{aligned}$$

Compared with traditional additive fusion, this multiplicative strategy provides stronger feature selectivity–only features that are simultaneously significant across the channel, temporal, and frequency dimensions receive high attention weights. This mechanism enables adaptive enhancement and suppression of features, allowing the model to focus on salient time–frequency regions and extract globally contextual representations that provide discriminative and robust support for subsequent classification or recognition tasks.

The global branch is composed of multiple stacked **GlobalAttention modules**, where the configuration of each stage differs in depth and channel dimensions. The upper-layer features are successively propagated to deeper layers as inputs, enabling a progressive transition from low-level representations to high-level global semantic features. In the experimental setup, the network is divided into four stages. Based on ablation studies and analysis of model parameter efficiency, the layer configuration is set to [2, 3, 3, 2], with corresponding convolutional channel numbers of [16, 32, 48, 64]. This configuration achieves an optimal balance between classification accuracy and model complexity. Such a design allows the model to rapidly capture global time–frequency patterns at lower dimensions in the initial stage and progressively expand feature channels in subsequent stages to enhance representational capacity.

As shown in Fig. 1 In the first stage, the input feature map is processed by two GlobalAttention modules for shallow feature extraction, primarily focusing on the basic energy distribution and short-term temporal dynamics of the audio signal. The second and third stages each stack three modules, introducing higher-dimensional convolutional channels (32 and 48, respectively) to jointly capture mid-level semantic structures across the temporal and frequency domains, such as rhythmic patterns and frequency-band responses. The final stage contains two GlobalAttention modules with an expanded channel width of 64, enabling the extraction of global contextual dependencies and high-level semantic representations that provide rich feature support for classification and recognition tasks.

Within each stage, the overall module architecture remains consistent, while specific parameters–such as the number of convolutional kernels, channel dimensions, and attention weight ratios–are adaptively adjusted according to the network depth, maintaining a balance between representational power and computational efficiency. Between stages, downsampling operations (e.g., strided convolution or pooling) are applied to achieve progressive compression of feature scales, allowing the model to form hierarchical multi-scale perception across both temporal and spectral dimensions. Through this hierarchical stacking structure, the model effectively aggregates global contextual information while maintaining lightweight characteristics, thereby generating more discriminative and robust time–frequency feature representations.

**Fig. 3:**
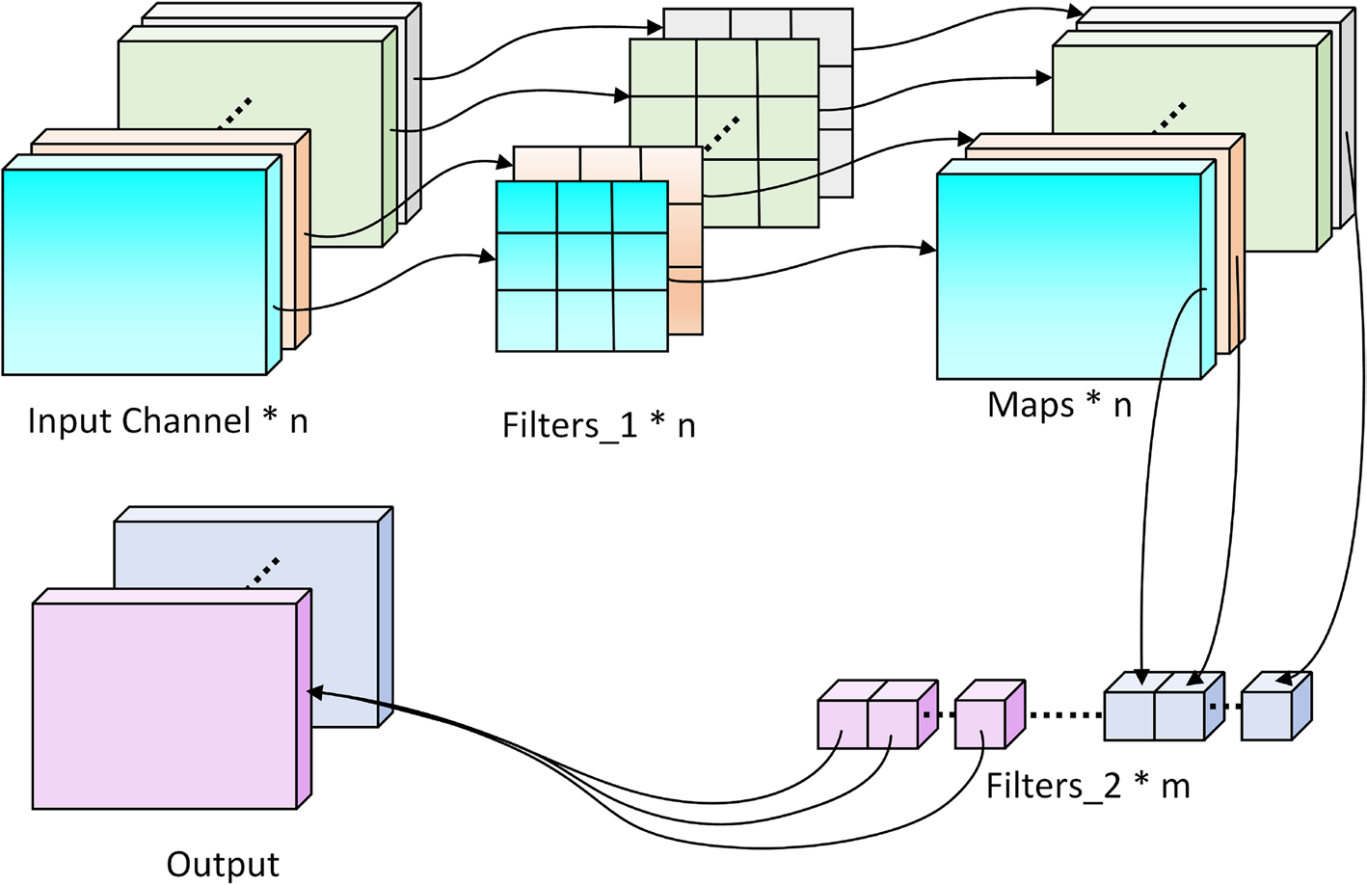
The DSC consists of two stages: depthwise convolution for spatial feature extraction within each channel and pointwise ($1\times 1$) convolution for channel fusion. Compared with standard convolution, it achieves comparable representational power with significantly reduced parameters and computational.

### Local branch design

In the local branch of SpectroMaskNet, we design and integrate a hierarchical local feature extraction framework composed of multiple stacked **LocalAttention** modules, as illustrated in Fig. 2. Each stage in the local branch follows the same layer configuration as the global branch, with layer numbers [2, 3, 3, 2] and corresponding channel dimensions [16, 32, 48, 64], enabling progressive feature refinement from shallow to deep levels. Unlike conventional convolutional neural networks (CNNs) that rely on repeatedly stacking small convolutional kernels to expand the receptive field, our LocalAttention module introduces a more efficient mechanism for modeling long-range local dependencies while maintaining computational efficiency.

The LocalAttention module is specifically designed to address three key challenges in lightweight acoustic modeling: (1) limited receptive field coverage caused by small convolutional kernels, (2) insufficient channel adaptivity for emphasizing informative frequency regions, and (3) reduced nonlinear representation capacity due to aggressive parameter compression. To tackle these issues, LocalAttention combines the receptive-field expansion strategy from ConvNeXtV2^26^ with the efficient feature transformation mechanism of NAFNet^27^. Structurally, each module consists of a large-kernel ($7\times 7$) depthwise separable convolution(DSC) to capture broad time-frequency context, followed by a Simplified Channel Attention (SCA) block for adaptive channel recalibration, a Local Feature Enhancement (LFE) bottleneck for nonlinear feature refinement, and a gating mechanism for adaptive information control.

Multiple LocalAttention modules are stacked across four hierarchical stages, where each stage operates at progressively coarser time-frequency resolutions through downsampling. This multi-stage structure allows the local branch to gradually expand its local receptive field while maintaining parameter efficiency. Finally, the local branch output is fused with the global branch output using a learnable weight coefficient $\lambda$, enabling the model to adaptively balance global contextual and local discriminative representations. Through this design, the local branch of SpectroMaskNet effectively captures fine-grained acoustic cues while remaining computationally lightweight and scalable.

At its core, the module adopts a large-kernel (*e.g.*, $7\times 7$) depthwise separable convolution (DSC) structure,as shown in Fig. 3. By decomposing standard 2D convolution into depthwise and pointwise operations, LocalAttention significantly reduces computational complexity and parameter count while capturing broader local time–frequency context in a single operation. Given the input feature map $X \in \mathbb {R}^{C\times T\times F}$, the process can be formulated as:

5 $$\begin{aligned} Y = W_p * (W_d * X), \end{aligned}$$

where $W_d$ and $W_p$ denote the depthwise and pointwise convolution kernels, respectively. This enables efficient aggregation of wide-range local dependencies within the spectrogram.

**Fig. 4:**
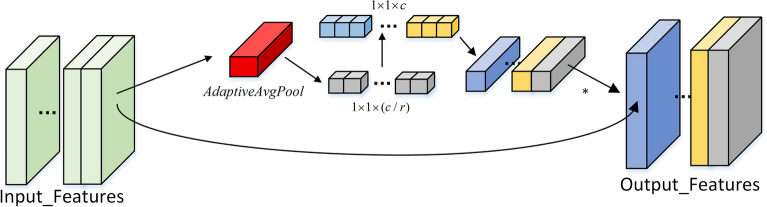
The structure of the simplified channel attention (SCA) module in the LocalAttention block.

To achieve channel-wise adaptive weighting and recalibration, we integrate a Simplified Channel Attention (SCA) mechanism, as shown in Fig. 4.

It first extracts global channel descriptors via global average pooling and then generates channel weights through two $1\times 1$ convolutions followed by Sigmoid activation:

6 $$\begin{aligned} s = \sigma (W_2\,\delta (W_1\,\text {GAP}(Y))), \end{aligned}$$

where $\delta (\cdot )$ and $\sigma (\cdot )$ represent the ReLU and Sigmoid functions, respectively, and $W_1$, $W_2$ correspond to reduction and expansion mappings. The resulting weights adaptively enhance informative channels while suppressing redundant ones.

To further enrich nonlinear expressiveness and strengthen local sensitivity, a Local Feature Enhancement (LFE) structure is embedded, which expands and restores channels through $1\times 1$ convolutions while modeling fine-grained dependencies via depthwise convolutions. Additionally, a gating mechanism is introduced to dynamically control the flow of information. The gate output, obtained through a $1\times 1$ convolution and Sigmoid activation, modulates the enhanced feature map through element-wise multiplication:

7 $$\begin{aligned} \hat{Y} = \sigma (W_g * Y) \odot Y, \end{aligned}$$

where $W_g$ denotes the gating convolution kernel and $\odot$ indicates element-wise multiplication. To ensure stable training and facilitate gradient propagation, Layer Normalization is applied before the main transformations, and the entire module is embedded within a residual connection, where the input is added to the output to mitigate gradient vanishing and support deeper stacking.

In summary, the LocalAttention module achieves an efficient integration of *large-kernel convolution*, *channel attention*, and *gated residual fusion*. This design enables the network to capture wide local time–frequency context and adaptively modulate feature responses under a lightweight framework, effectively overcoming the limitations of conventional convolutions in modeling long-range local dependencies. Consequently, LocalAttention enhances the discriminative power and robustness of SpectroMaskNet in complex acoustic environments while maintaining computational efficiency.In summary, the proposed training and inference pipeline for SpectroMaskNet is shown in Algorithm 1.

**Algorithm 1:**
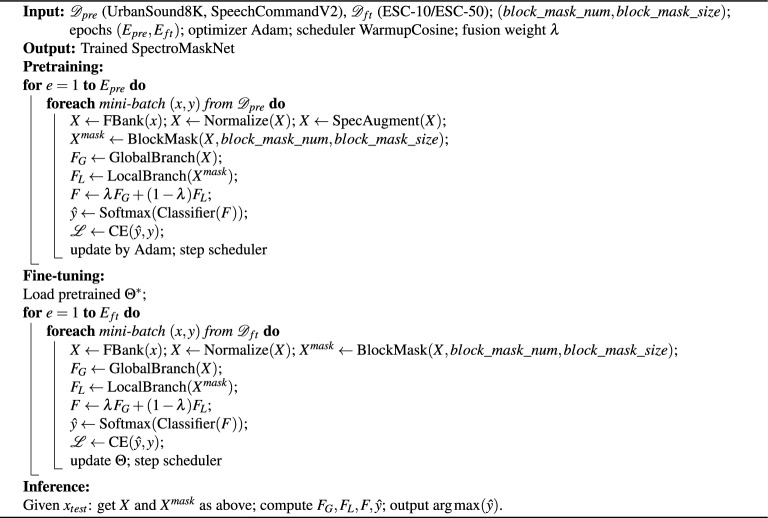
Training and inference of SpectroMaskNet (correct pretraining loop).

### Overall framework visualization and algorithm

After describing the detailed architecture of the global and local attention branches, we further summarize the overall workflow of the proposed SpectroMaskNet in Algorithm  1.. The algorithm outlines the complete process from spectrogram input, dual-branch feature extraction, cross-branch weighting, to final classification.

To gain deeper insight into the model’s internal attention behavior, we visualize the learned attention heatmaps of both the global and local branches in Fig. 6. As shown in the figure, the global branch primarily focuses on long-range contextual structures distributed across the time–frequency plane, such as global energy contours and temporal evolution patterns that are relevant to category-level semantics. In contrast, the local branch emphasizes fine-grained discriminative regions, including transient structures and localized spectral variations. These attention patterns are not random; instead, they emerge from the model’s adaptive learning process. The global branch captures holistic semantic cues, while the local branch concentrates on detail-rich spectral components, forming a complementary representation mechanism.

**Fig. 5:**
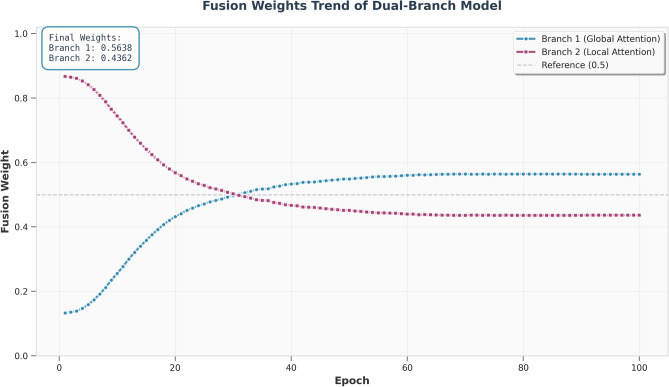
Training dynamics of the learnable fusion weights for the global-attention branch and the local-attention branch. The evolving weights illustrate how the model gradually adjusts the contribution of each branch, revealing their complementary roles during different stages of learning.

In addition, Fig. 5 presents the training dynamics of the learnable fusion weights between the two branches. When combined with the attention visualizations, these results help explain why the model attends to specific regions. During early training, the model relies more heavily on the local branch to quickly acquire sharp, fine-grained cues. As training progresses, the weight gradually shifts toward the global branch, indicating an increased reliance on stable contextual semantics. The coordinated evolution between the attention distribution and the fusion weights provides a coherent explanation for how the complementary global–local learning mechanism emerges.

Overall, these visual and quantitative analyses offer intuitive and theoretically consistent evidence that the dual-branch structure enables complementary feature learning, where the global branch models high-level semantic information and the local branch refines discriminative spectral details, ultimately enhancing the model’s classification capability.

**Fig. 6:**
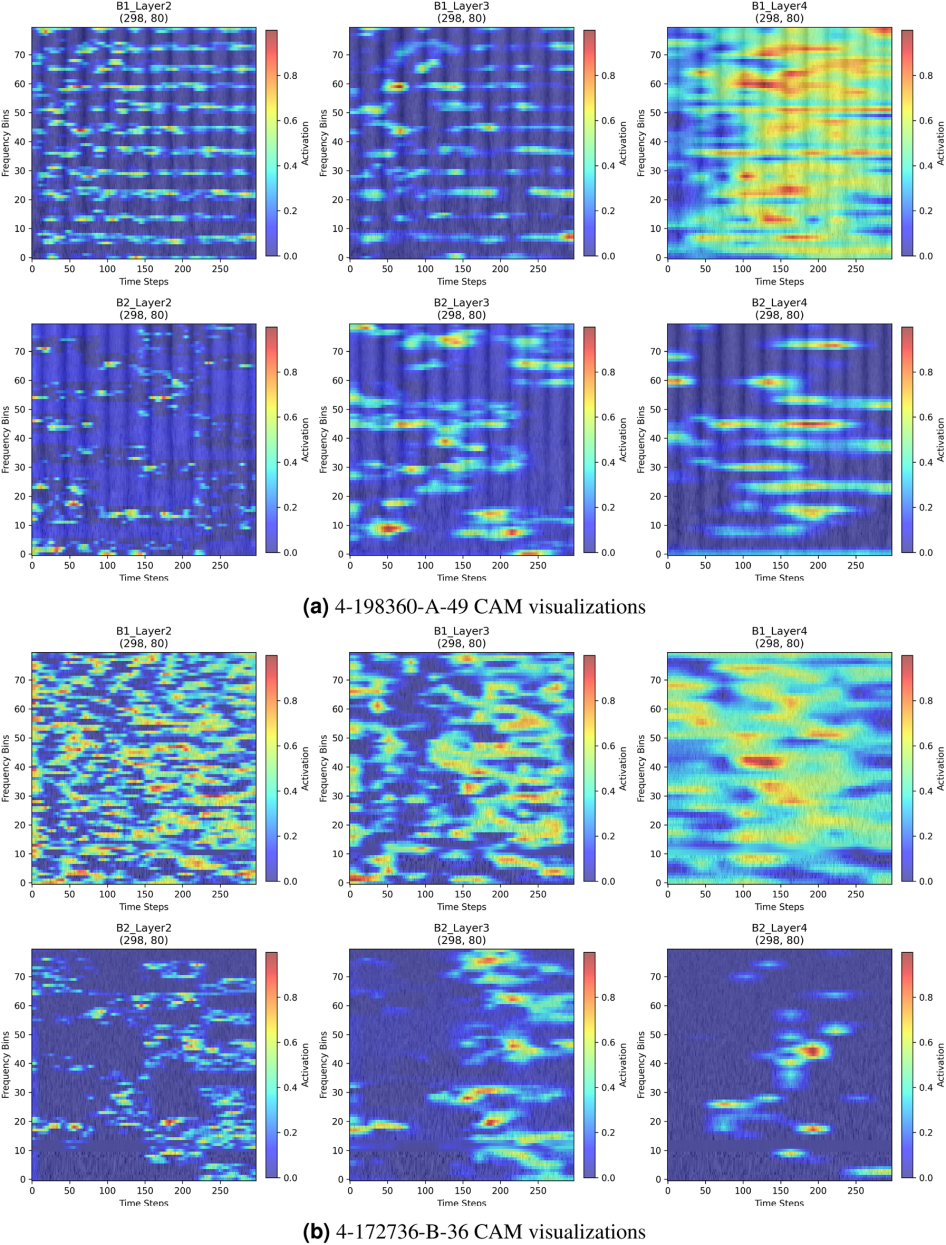
In the Speech Commands V2 dataset, CAM visualizations are shown for two samples across different attention layers. The first row corresponds to Branch 1, which takes the complete input and applies global attention; the second row represents Branch 2, where the input is masked and local attention is applied. As illustrated, distinct attention patterns emerge across layers and branches in the time-frequency domain. These visual differences highlight the model’s ability to capture multi-scale and multi-perspective acoustic features through a dual-branch attention architecture.

## Experiments

We conducted experiments on the ESC-10, ESC-50, UrbanSound8K, and SpeechCommandV2 datasets to investigate the impact of different masking rates and block sizes on the model’s generalization ability. By adjusting the masking input configurations, we explored the effect of masking strategies on model performance. Additionally, we examined the relationship between masked inputs and the attention mechanism by modifying the weight for feature fusion between the global and local branches. Finally, we used ESC-50, UrbanSound8K, and SpeechCommandV2 as pretraining datasets and fine-tuned the model on the ESC-50 dataset. The experimental results demonstrate that pretraining with small-scale datasets can significantly improve the model’s accuracy. Detailed experimental settings and analysis results can be found in the Ablation Study section.

### Datasets

In this experiment, we used several commonly used datasets in environmental sound classification (ESC) tasks to evaluate the performance of SpectroMaskNet. The main datasets used include ESC-10, ESC-50, UrbanSound8K, and SpeechCommandV2.

- **ESC-10** is a standard dataset for environmental sound classification, containing 10 different categories of environmental sounds, including both natural and human activity sounds. Each category contains approximately 400 audio samples, totaling around 4000 audio samples.
- **ESC-50** dataset is a complex environmental sound dataset containing 50 categories and a total of 2,500 audio samples, with 40 audio clips in each category. This dataset is widely used in environmental sound classification research and serves as an important benchmark for evaluating model generalization ability and feature extraction performance. Without any pre-training, the LHGNN^28^ model currently achieves the highest performance, with 33 million (33M) parameters and a top-1 accuracy of 96.2% on the ESC-50 dataset. Following closely, the M2D/0.7^29^ model attains a top-1 accuracy of **96.0%** under the same conditions but requires 86 million (86M) parameters.
- **UrbanSound8K** is a dataset of urban environmental sounds containing 8,000 audio samples from 10 categories. The samples represent various urban sound events, typically between 1-4 seconds in length.
- **SpeechCommandV2** is a dataset for speech command recognition, containing 12 basic speech commands and background noise samples. It is widely used for voice command classification and recognition tasks.

**Fig. 7:**
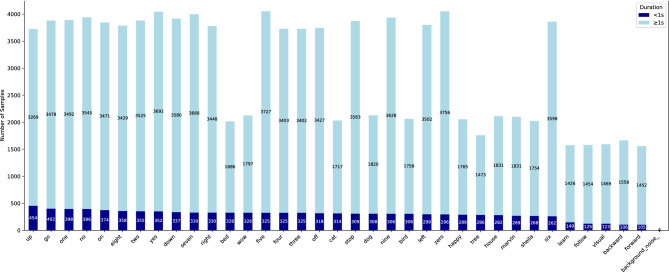
Duration distribution of audio samples across different classes in the speech commands V2 dataset.

In the subsequent experiments, we primarily focused on conducting classification experiments on the ESC-10 and ESC-50 datasets to evaluate the model’s performance in environmental sound classification tasks of varying scales. Meanwhile, the UrbanSound8K and SpeechCommandV2 datasets were mainly used for small-scale pretraining, serving as auxiliary datasets to enhance the model’s initialization. By pretraining on these two datasets and then fine-tuning on ESC-10 and ESC-50, we were able to significantly improve the model’s learning ability and generalization. Pretraining on small-scale datasets helps to reduce computational resource consumption and shorten training time, while also improving the model’s performance, particularly in low-data tasks. The combination of pretraining and fine-tuning strategies effectively boosts the model’s classification accuracy on specific tasks, prevents overfitting, and enhances the model’s stability and adaptability in resource-constrained real-world applications.

### Pretraining strategy

To improve model generalization under limited data conditions, we employ a lightweight pretraining–fine-tuning strategy^30^. Specifically, SpectroMaskNet is pretrained on two medium-sized datasets, UrbanSound8K and SpeechCommandV2, which contain diverse acoustic categories and sufficient temporal-spectral variation to learn transferable representations. Compared to large-scale corpora such as AudioSet, these datasets significantly reduce pretraining time and GPU memory consumption while maintaining feature diversity.

During pretraining, audio features are extracted as 80-dimensional FBank representations sampled at 16 kHz. The model is trained for 80 epochs using the Adam optimizer with an initial learning rate of $1\times 10^{-4}$ and a batch size of 8. All parameters are updated, and the classification head is reinitialized before fine-tuning. The pretrained weights are then transferred to downstream ESC datasets (ESC-10 and ESC-50) and fine-tuned for 60 epochs.

This strategy enables the model to learn general low- and mid-level spectral-temporal features from moderate-scale datasets, leading to a 5% accuracy improvement compared with models trained from scratch, while substantially reducing pretraining cost.

### Data preprocessing

In the UrbanSound and Speech Commands V2 datasets, variations in speech durations lead to time–frequency representations with inconsistent lengths along the time axis. To enable mini-batch training and parallel computation, a dynamic padding strategy is adopted: within each batch, samples are sorted in descending order based on sequence length, and zero-padding is applied along the time dimension to align all samples to the length of the longest one in the batch. Meanwhile, the original length of each utterance is preserved for subsequent use in masking operations or during CTC loss computation.

As illustrated in Fig. 7, the Speech Commands V2 dataset exhibits significant variation in sample durations across different classes. Although most samples are longer than 1 second (light blue bars), there still exists a non-negligible number of short utterances (<1s, dark blue bars) in each class. For example, the up, go, and one classes each contain more than 400 short-duration samples, whereas classes such as visual, forward, and backgroundNnoise have relatively fewer total samples.

### Feature extraction methods

**Fig. 8:**
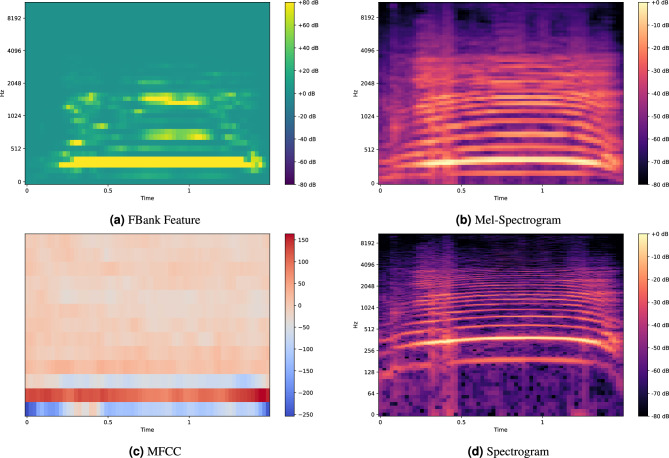
Visualization of different feature extraction methods on ESC-1-16568-A-3.wav Audio: The FBank feature extraction method, compared to Mel-Spectrogram, MFCC, and Spectrogram, has lower computational complexity and stronger robustness. It extracts the energy information of audio using the Mel filter bank without the complex logarithmic transformation and discrete cosine transform (DCT), making it more efficient for processing large-scale data. At the same time, FBank better preserves the spectral information in the low-frequency range and can effectively capture subtle differences in audio, especially in noisy environments. Therefore, it performs more stably in environmental sound classification and speech recognition tasks.

In this study, we chose to use FBank (filter bank) as the audio feature extraction method, rather than other common preprocessing methods such as MelSpectrogram, MFCC, or Spectrogram, based on the following considerations. Firstly, FBank features retain more spectral information compared to MelSpectrogram and MFCC, which is crucial for environmental sound classification tasks, as it captures subtle differences in audio signals. According to the results in the Table 1, using FBank features shows excellent classification performance on several datasets (such as ESC-10, ESC-50, UrbanSound8K, and SpeechCommandV2), with FBank achieving 97.50% on the ESC-10 dataset, significantly outperforming other feature extraction methods. Secondly, FBank features achieve high accuracy rates of 96.32% and 97.89% on the UrbanSound8K and SpeechCommandV2 datasets, respectively, further proving its stability and efficiency across different datasets. Moreover, FBank features exhibit strong robustness to noise, effectively handling complex environmental noise and multiple sound sources, thereby improving the model’s generalization ability. Visualization of various feature extraction methods is shown in Fig. 8. Additionally, the feature extraction process for FBank is relatively simple and computationally efficient, and its extraction formula is as follows:

8 $$\begin{aligned} F_m(t) = \sum _{f} \left| X(t, f) \cdot h_m(f) \right| \end{aligned}$$

where $F_m(t)$ is the output of the $m$-th filter bank, $X(t, f)$ is the spectrum of the audio signal, and $h_m(f)$ is the frequency response of the filter bank.In contrast, the MFCC feature extraction process involves multiple steps, including logarithmic transformation and discrete cosine transform (DCT), and its formula is as follows:

9 $$\begin{aligned} MFCC_m(t) = \sum _{n=1}^{N} \log \left( \sum _{f} \left| X(t, f) \cdot h_n(f) \right| \right) \cdot \cos \left( \frac{m(n-1)\pi }{N} \right) \end{aligned}$$

As seen from the above formulas, the MFCC feature extraction process is more complex compared to FBank, requiring additional logarithmic transformation and discrete cosine transform (DCT), which increases computational complexity and time costs. This makes the FBank feature extraction method more efficient when processing large-scale datasets. Therefore, choosing FBank as the feature extraction method allows for better capture of information in the audio signal, enhancing the overall performance of the model.

The FBank feature extraction method, compared to Mel-Spectrogram, MFCC, and Spectrogram, has lower computational complexity and stronger robustness. It extracts the energy information of audio using the Mel filter bank without the complex logarithmic transformation and discrete cosine transform (DCT), making it more efficient for processing large-scale data. At the same time, FBank better preserves the spectral information in the low-frequency range, and can effectively capture subtle differences in audio, especially in noisy environments. Therefore, it performs more stably in environmental sound classification and speech recognition tasks.

**Table 1:** The impact of different feature processing on classification results.

PreTrainingModel	DataSet	FeatureMethod			
		MelSpectrogram	Spectrogram	MFCC	Fbank
None	**ESC-10**	96.47	94.13	96.91	**97.50**
	**ESC-50**	92.54	94.18	93.21	**95.50**
	**UrbanSound8K**	93.89	91.83	95.32	**96.32**
	**SpeechCommandV2**	95.87	96.91	**97.89**	97.52

### Hyperparameter settings

In this study, audio feature extraction is performed using the Fbank (Filterbank) method. To improve the model’s performance in low-data scenarios, we pretrain the model on the UrbanSound8K and SpeechCommandV2 datasets. During the pretraining process, audio features are extracted using a 16,000 Hz sampling rate and 80 Mel filters. This pretraining allows the model to effectively learn low-level spectral features, which are crucial for environmental sound classification tasks, especially when data is limited. The learning during the pretraining phase enables the model to better adapt to specific tasks during subsequent fine-tuning, significantly improving its generalization ability and accuracy.

Additionally, during training, the audio data undergoes volume normalization to a target volume of -20 dB, ensuring consistency in the training data. A batch size of 8 is used during training, while a batch size of 4 is used for evaluation, with appropriate adjustments to the audio sample length. To enhance the model’s robustness, we also applied SpecAugment data augmentation, which involves frequency and time masking operations to further improve the model’s adaptability to noise and temporal variations.

For the optimizer, we used the Adam optimizer and combined it with the WarmupCosineSchedulerLR learning rate scheduler to adjust the learning rate gradually during training, ensuring better convergence. Label smoothing (label smoothing = 0.1) was applied to reduce overfitting, ensuring better generalization. To improve training efficiency and reduce computational resource consumption, this study adopts the pre-training and fine-tuning (Pre-training + Fine-tuning) approach. First, pre-training is conducted on a smaller dataset to leverage the generic features learned from large-scale datasets for initialization. Then, the model is further optimized during the fine-tuning phase to enhance its performance on the target task. The pre-training and fine-tuning approach not only accelerates the training process but also significantly improves the model’s generalization ability, especially when data is limited. After pre-training on different datasets and 150 rounds of fine-tuning, SpectroMaskNet demonstrated excellent performance on multiple environmental sound datasets, particularly in resource-constrained environments, where the model efficiently completed training and inference tasks.

### Model performance analysis

To validate the feasibility of our model in real-world deployment scenarios, we evaluated the inference latency and memory consumption of SpectroMaskNet on edge devices, as shown in Table 2. The results show that, even under the resource-constrained ARM architecture, the proposed model maintains low parameter counts and efficient inference performance, outperforming most baseline models. This advantage primarily stems from its lightweight dual-branch design and the use of efficient attention mechanisms, which jointly provide higher throughput and better energy efficiency in edge environments. In future work, we plan to further reduce the computational and storage overhead of the model through techniques such as pruning and quantization.

**Table 2:** Estimated inference latency and memory usage of different models on the Orange Pi 5B platform. The results demonstrate that the proposed SpectroMaskNet achieves one of the lowest latencies while maintaining moderate memory consumption, indicating strong suitability for real-time edge deployment.

Model	Inference Latency(ms)	Memory Usage(MB)
ResNetSE_Fbank	171	298
ECAPA-TDNN	**45**	265
PANNs-CNN10_Fbank	80	265
ERes2Net_Fbank	170	288
CAM++	73	305
Ours (SpectroMaskNet)	**45**	305

### Ablation study

To further validate the effectiveness of the proposed masking strategy and dual-branch attention mechanism, we conducted a series of systematic ablation experiments on the ESC-50 dataset. These experiments were designed to investigate the impact of the input masking mechanism and the global/local attention modules on the model’s performance under different configurations.

#### Analysis of the impact of masking strategy, masking frequency, and block size

To investigate the impact of masking block size and masking ratio on model performance, we first conducted a systematic set of ablation experiments on the ESC-50 dataset. In this part of the study, both the masking block size and the masking ratio were treated as fixed hyperparameters. The model was evaluated under different masking configurations, and the corresponding classification accuracy was analyzed. The results are summarized in Table 3.

**Fig. 9:**
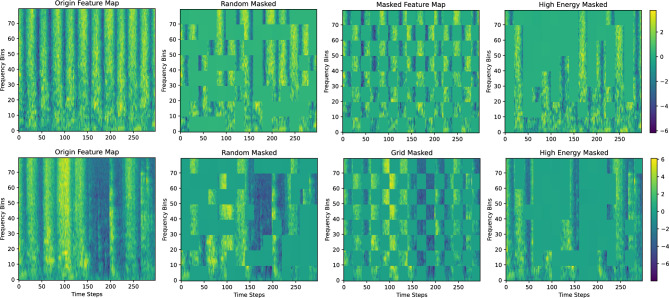
Comparison of spectrogram masking strategies. From left to right: original feature map, random masking, grid masking, and high-energy masking.

In our experiments, we adopted three different masking strategies: High-Energy Suppression Masking, Random Masking, and Grid Masking.Visualization of different masking strategies is shown in Fig. 9. High-Energy Suppression Masking is a guided masking method based on spectral energy analysis. By examining the energy distribution of the FBank-derived feature maps, this method selectively suppresses high-energy regions to reduce the model’s over-reliance on dominant components, thereby improving robustness under noisy or interfering conditions. Random masking discards local time–frequency patches with a fixed probability in a completely stochastic manner, introducing unbiased perturbations that help prevent overfitting. In contrast, grid masking applies structured square-shaped masking blocks across the feature map following a regular spatial pattern, imposing a stronger structural inductive bias on the learned representations.

**Table 3:** Ablation study on different masking strategies, showing the effect of block size (a), mask ratio (b), and mask strategy (c) on accuracy.The ablation results show that an appropriate masking strategy significantly improves model performance. Among the strategies, random masking outperforms high-energy and grid masking, likely due to its better input augmentation. A 50% masking ratio achieves optimal performance, aligning with findings in Masked Autoencoders (MAE). Medium-sized blocks (10$\times$10) yield the best results, balancing local and global information disruption.

a. Effect of masking block size		b. Effect of masking mask ratio		c. Effect of masking mask strategy	
Block Size	Accuracy (%)	Mask Ratio	Accuracy (%)	Mask Strategy	Accuracy (%)
4 $\times$ 4	89.5	0.3	87	**Radom**	**91.5**
8 $\times$ 8	90.7	**0.5**	**91.5**	High-energy	86.5
**10** $\times$ **10**	**91.5**	0.7	88.5	Grid	89.8
15 $\times$ 15	86.2	0.9	86.3		

Building upon the above experiments with fixed masking parameters, we further explored an adaptive masking mechanism designed to enhance the flexibility and task-specific suitability of the masking process. In this extended setting, the masking block size and masking ratio are no longer manually predefined; instead, they are formulated as learnable parameters and optimized jointly with the network weights through backpropagation. During training, these parameters automatically adjust their values: for example, the masking ratio tends to increase during early training to strengthen perturbation and encourage the learning of robust representations, and gradually decreases as training stabilizes. The block size exhibits a similar dynamic pattern–expanding during the exploratory phase and later converging to an optimal spatial range. These dynamic behaviors indicate that, compared with static masking configurations, the adaptive masking mechanism can regulate masking strength and coverage based on the model’s learning state, thereby providing more targeted regularization during feature learning.

**Fig. 10:**
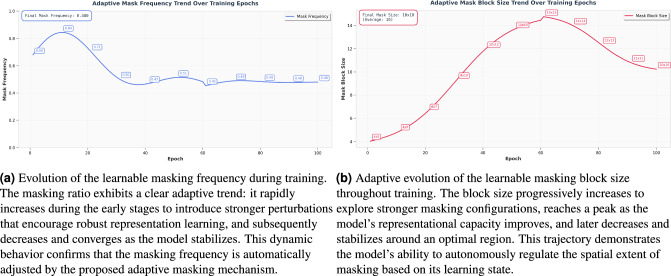
Evolution of the learnable masking frequency and masking block size during training.

Across both the fixed-parameter ablation study and the adaptive masking mechanism, the model converges to very similar optimal masking configurations. In the fixed-setting experiments, a block size of 10$\times$10 and a masking ratio of 0.5 yield the highest accuracy (91.5%). Remarkably, the adaptive masking strategy–where both parameters are learned end-to-end–naturally evolves toward nearly the same values during training, as shown in Fig. 10. This consistency suggests that the selected masking configuration represents an intrinsic optimum for balancing information removal and feature preservation. Moreover, it indicates that the model is capable of autonomously discovering this optimal masking pattern through gradient-based learning, reinforcing the validity of our masking design and confirming the robustness of the proposed SpectroMaskNet across both manual and adaptive configurations.

#### Comparative analysis of global and local attention module substitutions.

To demonstrate the impact of different branch input configurations and attention mechanisms on the performance of audio classification models, two main branch configurations were adopted in the experiment: First, the global branch processes the original FBank features, while the local branch processes the masked FBank features. Second, the inputs of the two branches were swapped, with the global branch processing the masked FBank features and the local branch processing the original FBank features. To further evaluate the effect of the attention mechanism, this study also compared the use of the SE attention mechanism with no attention mechanism, and observed the accuracy changes of the model on the ESC-50 dataset. Through these settings, the experiment aims to quantify the contribution of different branch inputs and attention mechanisms to the model’s performance, revealing the key role of global and local feature processing as well as attention weighting in audio feature learning.For comparative validation, we replaced the aforementioned global-local attention with the standard Squeeze-and-Excitation (SE) module, or completely removed the attention mechanism while retaining only the convolutional backbone. The Table 4 show that the model performs best when using the full global-local attention structure, with a slight performance decrease when replaced by the SE module, and a significant drop in model generalization ability when the attention mechanism is completely removed.

**Table 4:** Comparison of classification accuracy under different attention mechanisms and input configurations (No mask and Mask). The table presents the accuracy results for various attention mechanisms, including G-Attention, L-Attention, SE-Attention, and No Attention, evaluated on both unmasked and masked input features.

input	No mask	Mask	Accuracy (%)
**Attention**	**G-Attention**	**L-Attention**	**91.5**
	L-Attention	G-Attention	88.5
	SE-Attention	SE-Attention	87.5
	No Attention	No Attention	86.1

#### Pre-training influence

Large-scale dataset pre-training typically requires a long training time and significant computational resources. To achieve a more efficient training process, this study chose two smaller-scale audio datasets, UrbanSound8K and SpeechCommandV2, for pre-training. As shown in Table 5 When pre-trained on the UrbanSound8K dataset, the model achieved accuracy rates of 97.5% and 95.5% on the ESC-10 and ESC-50 datasets, respectively. When pre-trained on the SpeechCommandV2 dataset, the model achieved accuracy rates of 97.5% and 93% on ESC-10 and ESC-50. In contrast, models with no pre-training (None) achieved accuracy rates of only 95% and 91.5% on ESC-10 and ESC-50, respectively, indicating that pre-training significantly enhances the model’s performance.

**Table 5:** The performance comparison of the dual-branch masking network pre-trained on small-scale datasets on ESC-10,ESC-50,UrbanSound8K and SpeechCommandV2.

Dataset	PretrainingDataset	PretrainEpoch	$\lambda$	BlockSize	Accuracy (%)
ESC-10	None	None	0.5	10	95
ESC-50					91.5
UrbanSound8k					95.23
SpeechCommandV2					**97.52**
ESC-10	UrbanSound8k	60	0.5	0.5	97.5
ESC-50					**95.5**
UrbanSound8k					95.23
SpeechCommandV2					97.61
ESC-10	SpeechCommandV2	15	0.5	0.5	**97.5**
ESC-50					93
UrbanSound8k					**96.14**
SpeechCommandV2					97.61

Compared to large-scale dataset pre-training schemes (such as ImageNet^31^ and AudioSet), pre-training on smaller-scale datasets not only reduces training time and computational costs but also aligns better with the lightweight model structure used in this study. Given the smaller parameter size of the model, the performance gains from pre-training may plateau on large datasets. However, pre-training on appropriately sized datasets can more effectively activate the model’s potential and improve the initialization of feature extraction. For instance, with pre-training on UrbanSound8K and SpeechCommandV2, the model converges quickly and shows significant improvement in accuracy. These results demonstrate the effectiveness of pre-training on smaller-scale datasets, ensuring training efficiency while laying a solid foundation for rapid convergence and performance improvement in the target task.

### Comparison experiments

#### Comparison of models with different parameter sizes on ESC-50

This study compares the performance of several audio classification models with different pre-training datasets and parameter sizes on the ESC-50 dataset. As shown in Table 6, in terms of the pre-training dataset scale, models trained on large-scale datasets (e.g., ImageNet + AudioSet) such as AST and PaSST-S show a significant advantage over models trained on smaller datasets (e.g., UrbanSound8k) such as Ours and TDNN. Specifically, AST and PaSST-S achieve accuracy rates of 95.6% and 96.8%, respectively, while Ours achieves an accuracy of 95.5%. Despite using a smaller pre-training dataset, Ours still delivers comparable performance.

**Table 6:** The comparison experiment of pre-training with different dataset scales and parameter sizes on ESC-50.

Model	PretrainingDataset	#Parameters($\times 10^{6}$)	Accuracy on ESC-50(%)
AST^2^	ImageNet+AudioSet	88.1	95.6
PaSST-S^3^	ImageNet+AudioSet	85.4	**96.8**
EAT^1^	AudioSet	88	95.9
EAT-M^32^	AudioSet	25.5	96.3
EAT-S^32^	AudioSet	5.3	95.25
TDNN^33^	UrbanSound8k	2.7	83.31
Res2Net^34^	UrbanSound8k	6.2	90.51
**Ours**	**UrbanSound8k**	**2.7**	**95.5**

Regarding parameter size, models with large parameter counts, such as AST and PaSST-S, have 88.1M and 85.4M parameters, respectively. Although these models perform excellently, their computational cost is relatively high. In contrast, Ours, a lightweight model with only 2.7M parameters, still achieves 95.5% accuracy on the ESC-50 dataset. This demonstrates that lightweight models can also achieve excellent performance with smaller datasets while significantly reducing computational resource consumption. When pre-training on UrbanSound8K using an A800-80G, Ours requires only 3 hours for pre-training and 0.5 hours for fine-tuning, whereas the EAT model requires 96 hours on the same hardware. Thus, while maintaining a high accuracy rate, Ours demonstrates significant advantages in computational efficiency and resource savings, highlighting its immense potential and superiority in real-world applications.

**Table 7:** The comparison experimental results of different pre-trained models on the ESC-10, ESC-50, and SPCv2 datasets.

PreTrainModel	Model	ESC-10	ESC-10	ESC-10	#Parameters
		Accuracy(%)	Accuracy(%)	Accuracy(%)	$\times 10^{6}$
None	TDNN^33^	90.12	78.51	95.41	2.7
	ResNetSE^35^	92.51	89.50	95.45	9.1
	ECAPA_TDNN^36^	89.58	81.25	96.45	6.4
	CAM++^37^	95.10	86.75	95.54	7.1
	Panns^38^	85.31	73.89	96.43	5.2
	**Ours**	**95.00**	**91.50**	**97.52**	**2.7**
UrbanSound8K	TDNN^33^	91.06	83.31	96.11	2.7
	ResNetSE^35^	93.81	90.05	95.78	9.1
	ECAPA_TDNN^36^	90.28	83.11	96.12	6.4
	CAM++^37^	96.39	84.24	95.82	7.1
	Panns^38^	88.05	78.91	96.98	5.2
	**Ours**	**97.50**	**95.5**	**97.61**	**2.7**

#### Comparison of lightweight models’ performance on different datasets after pretraining

In this experiment, we pretrained different lightweight models using the UrbanSound8K dataset and compared their performance on three datasets (ESC-10, ESC-50, SPCv2). The purpose of the experiment was to verify the feasibility of pretraining with a small-scale dataset and explore the adaptability of our proposed model in this task. As shown in Table 7 The results showed that the models pretrained on UrbanSound8K performed significantly better during testing. For instance, on the ESC-10 dataset, the model pretrained with UrbanSound8K, Ours, achieved an accuracy of 97.50%, a notable improvement compared to the model that was not pretrained (95.00%). On the ESC-50 dataset, Ours reached 95.50%, and on the SPCv2 dataset, the accuracy rose to 97.61%. These results strongly demonstrate that even a relatively small dataset like UrbanSound8K can effectively provide support for transfer learning, improving model performance on other tasks.

Notably, our model’s adaptability across different datasets is especially impressive. Whether on the small-scale ESC-10 dataset or the relatively larger SPCv2 dataset, Ours pretrained on UrbanSound8K showed robust performance and achieved excellent results on multiple datasets.

In contrast, other models (such as TDNN and ResNetSE) also showed improvement after pretraining with UrbanSound8K, but their performance still lagged behind Ours. This indicates that our model has stronger adaptability for pretraining with small-scale datasets, demonstrating consistent and superior performance across tasks of varying scales. By incorporating a masking strategy with a global-local dual-branch approach, the model can efficiently extract features at multiple levels. The global branch captures the overall time-series information, while the local branch focuses on learning detailed features. This strategy allows the model to better balance global and local information on small-scale datasets. Moreover, the masking strategy further enhances the model’s robustness against noise and irrelevant information, improving its generalization ability in data-scarce scenarios. Therefore, the ResNetSE model pretrained on UrbanSound8K performs excellently across multiple datasets, showcasing its strong adaptability and efficient transfer learning capability on small-scale datasets.

## Conclusion

This paper presents SpectroMaskNet, a lightweight dual-branch network that integrates a global–local attention mechanism with a block-masked spectrogram augmentation strategy for environmental sound classification. The model effectively captures both global context and local spectral details while maintaining low computational complexity, enhancing robustness and generalization under limited-data conditions.

Extensive experiments on four benchmark datasets–ESC-10, ESC-50, UrbanSound8K, and SpeechCommandV2–demonstrate the effectiveness of the proposed approach, achieving accuracies of 97.5%, 95.5%, 96.32%, and 96.52%, respectively. On ESC-50, SpectroMaskNet reaches 95.5% accuracy with fewer than 3 million parameters, approaching large Transformer-based models such as AST (95.6%) and PaSST-S (96.8%), while requiring only about 1/30 of their computational cost. Compared with conventional lightweight CNNs (e.g., Res2Net, TDNN), it shows superior accuracy and generalization.

Moreover, to validate practical deployability, we evaluated SpectroMaskNet on actual edge hardware. The results show that the model maintains low memory consumption and achieves faster inference compared with several baseline architectures of similar or larger size, confirming its suitability for real-time processing in resource-constrained environments.

In summary, SpectroMaskNet achieves an excellent balance between efficiency and performance, making it highly suitable for real-world applications requiring compact, fast, and reliable sound recognition. Future work will explore self-supervised pretraining, temporal masking strategies, and hardware-aware optimization to further enhance robustness and real-time capability.

## Data Availability

The datasets used in this study are publicly available. The following are the links to the datasets: ESC-10: https://github.com/karolpiczak/ESC-10ESC-50; https://github.com/karolpiczak/ESC-50 urbansound8k: https://audeering.github.io/datasets/datasets/urbansound8k.html Speech Commands V2: https://aistudio.baidu.com/datasetdetail/222925/0.
